# Protein Interface Complementarity and Gene Duplication Improve Link Prediction of Protein-Protein Interaction Network

**DOI:** 10.3389/fgene.2020.00291

**Published:** 2020-04-02

**Authors:** Yu Chen, Wei Wang, Jiale Liu, Jinping Feng, Xinqi Gong

**Affiliations:** ^1^School of Mathematics, Renmin University of China, Beijing, China; ^2^School of Mathematics and Statistics, Minnan Normal University, Zhangzhou, China; ^3^Institute for Mathematical Sciences, Renmin University of China, Beijing, China; ^4^School of Mathematics and Statistics, Henan University, Kaifeng, China

**Keywords:** protein-protein interaction, network, link prediction, interface complementarity, gene duplication

## Abstract

Protein-protein interactions are the foundations of cellular life activities. At present, the already known protein-protein interactions only account for a small part of the total. With the development of experimental and computing technology, more and more PPI data are mined, PPI networks are more and more dense. It is possible to predict protein-protein interaction from the perspective of network structure. Although there are many high-throughput experimental methods to detect protein-protein interactions, the cost of experiments is high, time-consuming, and there is a certain error rate meanwhile. Network-based approaches can provide candidates of protein pairs for high-throughput experiments and improve the accuracy rate. This paper presents a new link prediction approach “Sim” for PPI networks from the perspectives of proteins' complementary interfaces and gene duplication. By integrating our approach “Sim” with the state-of-art network-based approach “*L*3,” the prediction accuracy and robustness are improved.

## 1. Introduction

Protein is the executor of all biological physiological functions, and most of the cell functions are accomplished by interactions of proteins. Therefore, the detection and prediction of protein-protein interactions is of great significance for understanding the mechanism of life activities. In recent years, with the development of biotechnology, some techniques for identifying protein-protein interactions have been developed, such as Yeast two-hybrid (Y2H) (Fields and Song, 1989), Co-Immunoprecipitation (Moresco et al., 2010), Affinity chromatography (Cuatrecasas, 1970), and Protein Chips (MacBeath and Schreiber, 2000). These techniques provide us with a large amount of data on protein-protein interactions. However, the experimental results are mixed with a large number of false positive and false negative data. Meanwhile, the cost of experiments is very high. Therefore, more and more scholars use computational methods to predict protein-protein interactions. At present, there are many computational methods based on genome information, genetic evolution (Tsoka and Ouzounis, 2000; Chen et al., 2006; Lin et al., 2013) and protein structure (Planas-Iglesias et al., 2013; Zhao et al., 2017). These methods explain the principle of protein-protein interactions from different aspects. However, the information needed by many of these methods is not easily obtained, so they are not of universal significance. Many sequence-based machine learning methods (Huang et al., 2016; An et al., 2017; Wang et al., 2017a,b; You et al., 2017) have been developed. Based on the primary sequences of proteins, they use machine learning algorithms, such as Neural Network (Wang et al., 2017b), Support Vector Machine (SVM) (Wang et al., 2017a), and rotation forest (You et al., 2017) to predict protein-protein interactions.

**Figure 1:**
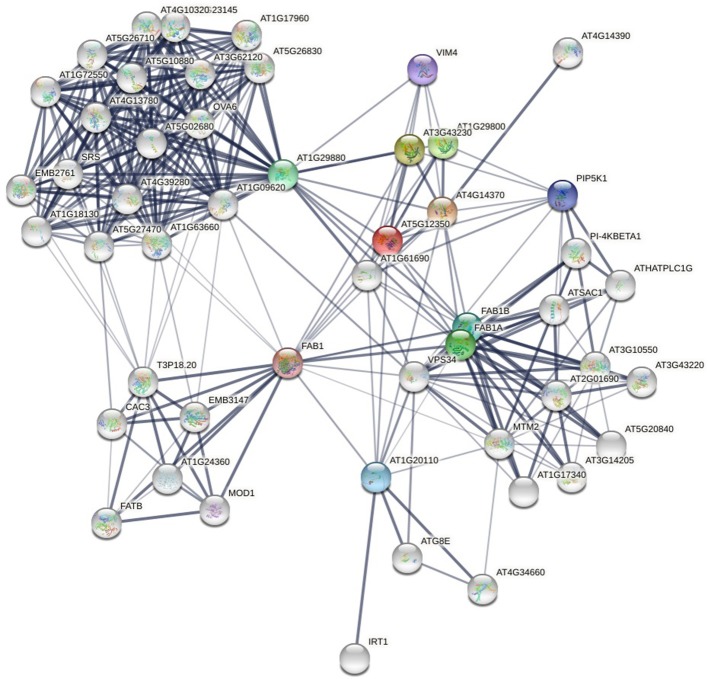
Protein-protein interaction network of yeast (a local part), from (STRING). The nodes are proteins and the links are interactions between them. The thickness of the edge represents the confidence of the interaction.

With the development of experimental and computational methods, protein-protein interaction data increase rapidly. There are many databases that store protein-protein interaction data. The PPI network (see Figure 1) formed by these interactions contains a lot of information. How to discover new links from the already known PPI networks has become a research hotspot in proteomics. Unlike stochastic networks, PPI networks have the characteristics of small-world networks, such as short average path length and power-law distribution of node degrees. These common characteristics have inspired scholars to study PPI networks in the way of studying social networks. These methodologies are mainly divided into three categories: neighborhood-based or paths-based approaches (Cannistraci et al., 2013; Huang et al., 2017; Muscoloni et al., 2018; Kovács et al., 2019; Pech et al., 2019), hierarchical clustering approaches (Clauset et al., 2008; Symeonidis et al., 2013), and random walk-based approaches (Lichtenwalter et al., 2010; Backstrom and Leskovec, 2011).

Network-based methods usually use common neighbors and paths between each pair of nodes to define “similarity” between them and use it to measure the link likelihood of them. These methods originate from the research of link prediction of social networks (Lü and Zhou, 2011; Wang et al., 2015). Their basic ideas are that two highly similar nodes (tightly connected through intermediate nodes) in a network tend to establish new links. For example, if two people who do not know each other yet have many friends in common, we predict that they may know each other in the future. That is to say, the number of common neighbors (the number of two-hop paths) (Newman, 2001) is related to the link likelihood between the two nodes. At the same time, the number of *k*-hop paths is also considered for the prediction of network links. The design of these similarity indices well reflects the link self-organization mechanism of some social networks, and some of them perform well on some PPI networks as well. We list several network-based indices (mostly based on common neighbors and paths), as shown in Table 1, where Γ_*i*_ is the neighbor set of node *i*, *k*_*i*_ is the degree of *i*, and *A* is the adjacency matrix.

**Table 1:** Indices for network-based approaches.

**Method**	**References**	**Index**	**Length of path**
CN (Common neighbor)	Newman (2001)	*CN*_*ij*_ = |Γ_*i*_⋂Γ_*j*_|	*l* = 2
JC (Jaccard index)	Jaccard (1912)	$JC_{ij}=\frac{\left|\Gamma_{i}⋂\Gamma_{j}\right|}{\left|\Gamma_{i}⋃\Gamma_{j}\right|}$	*l* = 2
LHN (Leicht-Holme-Newman)	Wang et al. (2015)	$LHN_{ij}=\frac{\left|\Gamma_{i}⋂\Gamma_{j}\right|}{k_{i}*k_{j}}$	*l* = 2
AA (Adamic-Adar)	Adamic and Adar (2003)	$AA_{ij}=\sum_{z\in \Gamma_{i}⋂\Gamma_{j}}\frac{1}{\log k_{z}}$	*l* = 2
PA (Preferential attachment)	Barabâsi et al. (2002)	*PA*_*ij*_ = *k*_i_*k_*j*_	Other
RA (Resource allocation)	Zhou et al. (2009)	$RA_{ij}=\sum_{z\in \Gamma_{i}⋂\Gamma_{j}}\frac{1}{k_{z}}$	*l* = 2
LP (Local path)	Lü et al. (2009)	$LP_{ij}={\left[A^{2}+\beta A^{3}\right]}_{ij}$	*l* = 2, 3
Katz	Katz (1953)	$Katz_{ij}={\left[\beta A+\beta^{2}A^{2}+\ldots \right]}_{ij}$	*l* = 1, 2, …

The advantages of network-based methods are of high efficiency (fast calculation speed, decent accuracy), easy access for inputs (Only PPI data is needed as inputs), and good generalization (applicable to all protein-protein networks). However, these indices, which are successful in predicting links of social networks, are not necessarily suitable for PPI networks (Kovács et al., 2019). The fundamental reason is that the self-organization mechanism of PPI networks is different from that of social networks. Furthermore, the above researches (Table 1) show that the number of short paths between two nodes in social networks has a greater impact on the link likelihood between them than the number of long paths. Because of that, these indices are usually based on 2-hop paths, or the impact on the index decreases with the increase of the path length, such as *Katz*. However, the principle that people tend to build relationships with people who are close to them in social networks cannot explain the interaction of two proteins. Therefore, some scholars (Muscoloni et al., 2018; Kovács et al., 2019; Pech et al., 2019) attempt to explain the link mechanism of PPI networks with 3-hop paths rather than 2-hop paths.

Starting from the demonstration of structure and evolution, Kovács et al. (2019) proposed a link prediction principle: predicting undiscovered protein interactions based on 3-hop paths (*L*3) (see Equation 1). They argue that two proteins sharing multiple interaction partners have similar interaction interfaces, and there is no reason to predict their interaction unless the interface can self-interact to form a homodimer. Experiments on many PPI networks show that their method outperforms indices based on 2-hop paths. For the first time, they confirmed that for PPI networks, the link likelihood between two nodes is more related to the paths of length 3 than the paths of length 2.

(1) $$\begin{array}{l} L3_{ij}=\underset{z_{1},z_{2}\in L3}{\sum }\frac{a_{iz_{1}}a_{z_{1}z_{2}}a_{z_{2}j}}{\sqrt{k_{z_{1}}k_{z_{2}}}} \end{array}$$

where *z*_1_, *z*_2_ are the intermediate nodes in the 3-hop path *L*3; *a*_*iz*_1__, *a*_*z*_1_*z*_2__, and *a*_*z*_2_*j*_ are the link strength of *iz*_1_, *z*_1_*z*_2_, and *z*_2_*j*, respectively.

Through a simple assumption that the possibility of a link between two nodes can be unfolded by the linear summation of the contributions of neighbors, Pech et al. (2019) proposed an optimization problem for similarity matrix *S* (i.e., Equation 2).

(2) $$\begin{array}{l} \underset{S}{\min}\alpha ||A-AS|{|}_{F}^{2}+||S|{|}_{F}^{2} \end{array}$$

where α is a free parameter, *A* is the adjacency matrix of network *G*, and *S* is the similarity matrix.

And they obtained the analytical solution of the optimal likelihood matrix S^*^, based on which their index: *LO* (Equation 3) shows better performance in predicting missing links than many other path-based methods. Since Kovács et al. (2019), they have confirmed once again that the number of 3-hop paths between two nodes is more useful for predicting the missing link between them than the number of 2-hop paths. Interestingly, an equivalent variant of *LO* shows a similar form to *Katz*.

(3) $$\begin{array}{l} LO=AS^{*}=\alpha A{\left(\alpha A^{T}A\;+\;I\right)}^{-1}A^{T}A=\left[\alpha A^{3}\;-\;\alpha^{2}A^{5}\;+\;\alpha^{3}A^{7}\;-\;\alpha^{4}A^{9}\;+\;\ldots \;\right] \end{array}$$

where S^*^ is the optimal likelihood matrix of Equation (2).

In summary, Kovács et al. (2019) illustrates the basis of their methods: Principle of *L*3 from the perspective of protein structure and evolution. Pech et al. (2019) assumes that the link likelihood of node *i* and *j* equals the linear combination of the similarity between *i*'s neighbors and *j*. And then they use linear optimization to obtain the optimal similarity matrix and the link likelihood matrix.

The index *L*3 can be written as:

(4) $$\begin{array}{l} L3_{ij}=\underset{z_{1}\in \Gamma \left(i\right)}{\sum }\underset{z_{2}\in \Gamma \left(z_{1}\right)\cap \Gamma \left(j\right)}{\sum }\frac{a_{iz_{1}}a_{z_{1}z_{2}}a_{z_{2}j}}{\sqrt{k_{z_{1}}k_{z_{2}}}} \end{array}$$

From Equations (3) and (4), we can see that, what *L*3 and *LO* have in common is that they both assume that the link probability of nodes *i* and *j* is equal to the linear combination of the similarities between *j* and *i*'s neighbors. Although the similarity matrix in *LO* does not need to be defined beforehand, the *LO* = AS^*^ obtained from Equation (2) can not necessarily achieve the optimal prediction effect. The reason is that the minimum of $||A-AS|{|}_{F}^{2}$ does not guarantee that the order of element values in *AS* is the most consistent with *A*. *L*3 can be regarded as a special case of *LO*. And the prediction ability of *L*3 is even greater than that of *LO* on some PPI networks. We will give some examples to illustrate this in section 2.4. Another disadvantage of *LO* is that there is no good way to determine the value of the free parameter α, it is just declared that α is a very small number in Pech et al. (2019). We set α = 0.00001 in this paper. There is still room for the improvement of the form of the index *AS*. Similarity matrix *S* is the key for the improvement. It is needed to propose a more reasonable definition of *S* which can be explained from the biological point of view.

## 2. Materials and Methods

### 2.1. Similarity Measure

We first study the similarity measure between two proteins. It is generally believed that the function of a protein is determined by its structure (Planas-Iglesias et al., 2013). However, compared with sequence information, information about the spatial (tertiary and quaternary) structure of proteins is scarce. There is a cost to determine the protein structure by technologies, such as X-ray and Cryo-EM. Although all kinds of deep learning algorithms [such as RaptorX (Peng and Xu, 2011) and Alpha Fold (AlQuraishi, 2019)] have greatly improved the accuracy of protein structure prediction, it is still an open problem, and the protein structure data grows slowly compared with sequence information. For any PPI network, we usually find that not all proteins have already known 3D structure and information of interaction interfaces. Therefore, there is no generalization significance in using protein structure information to predict PPI network links.

Network-based methods do not need structural information or even sequence information to predict links. The key to such methods is similarity measure. Similarity measure between two nodes in a network has been intensive studied (Jaccard, 1912; Katz, 1953; Newman, 2001; Barabâsi et al., 2002; Adamic and Adar, 2003; Lü et al., 2009; Zhou et al., 2009; Wang et al., 2015). However, most previous studies of link prediction suggested that the higher the similarity between two nodes, the more likely they are to be connected. It makes sense that in social networks, two people who have many common friends or interests are likely to become friends (Newman, 2001) or well-connected people attract each other (Barabâsi et al., 2002).

### 2.2. Why Jaccard Similarity?

Whether for social networks or PPI networks, the core idea of network-based link prediction methods we mentioned in section 2.1 is to design a similarity measure between nodes for their networks, which determines the likelihood of the linkage between each pair of nodes. Therefore, these similarity measures in Table 1 are directly used as indices of link prediction. There are different reasons for the selection of their similarity measure, respectively, such as Preferential Attachment (Barabâsi et al., 2002), Resource Allocation (Zhou et al., 2009), and Reciprocal Relationship (Dick and Green, 2018), etc. The index we are going to propose is still network-based, but there are two differences between our method and the previous ones in Table 1:

1. The similarity used in our index is Jaccard Similarity (Jaccard, 1912). In this subsection, we will explain the reasons for choosing Jaccard Similarity from two aspects.
2. Unlike the indices in Table 1, we do not directly use the Jaccard Similarity between node *u* and *v* to predict links between them, but use the linear combination of Jaccard Similarities between one node's neighbors and the other node. We will explain the reasons in this subsection and section 2.4.

There are two reasons for choosing Jaccard Similarity:

#### 2.2.1. Complementary Interfaces of Interacting Proteins

**Figure 2:**
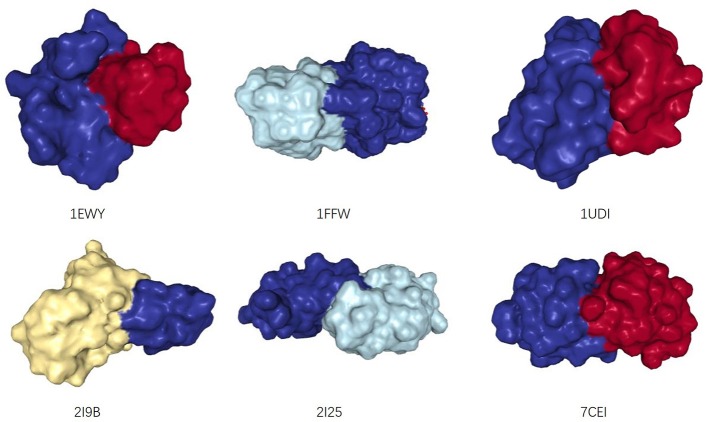
The structures of six dimers, from: (RCSB PDB). Two interacting monomers are represented by different colors. Their interaction interfaces are complementary.

For PPI networks, proteins with similar structures share similar interaction interfaces (Norel et al., 1994). Therefore, the two interacting proteins have complementary interfaces to each other (see Figure 2). In other words, two proteins with similar interfaces are likely to share more interacting neighbors rather than interacting with each other. The more similar they are, the greater proportion of common neighbors. We mentioned earlier that the structural information of proteins is not easy to obtain, but we can infer the similarity between them by the proportion of their common neighbors, i.e., Jaccard Similarity (Jaccard, 1912).

(5) $$\begin{array}{l} J_{ij}=\frac{\left|\Gamma_{i}\cap \Gamma_{j}\right|}{\left|\Gamma_{i}\cup \Gamma_{j}\right|} \end{array}$$

where Γ_*i*_ is the set of neighbors of node *i* in the PPI network.

**Figure 3:**
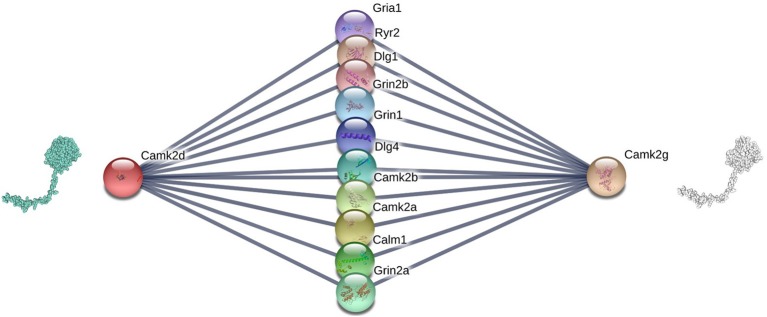
Camk2d, Camk2g, and their neighbors in the PPI network of Rattus norvegicus, from: (STRING). The similarity of their interaction interfaces determines that they can share a large proportion of interaction partners.

**Figure 4:**
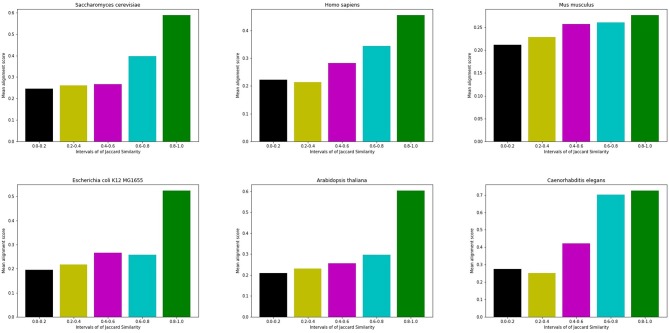
Mean alignment score in each interval of Jaccard Similarity. On the whole, the alignment score of high Jaccard Similarity node pairs is higher than that of low Jaccard Similarity node pairs.

Figure 3 shows an example of the correlation between interface similarity and Jaccard Similarity. We can see from the naked eye that Camk2d and Camk2g have similar 3D structure, which leads to their similar interaction interfaces. Therefore, they may share a large proportion of interaction neighbors who have complementary interfaces to them, respectively, i.e., Jaccard Similarity. Since not every protein in PPI network has already known 3D structure, we use the global alignment algorithm (Needleman and Wunsch, 1970) to measure the similarity between protein pairs. The alignment score of Camk2d and Camk2g is 0.83395, which means they have very similar amino acid sequences. Furthermore, we also found evidence in the database (PhylomeDB) that they are paralogues derived from gene duplication events. Figure 4 shows the mean pairwise alignment score in each interval of Jaccard Similarity. With the increase of Jaccard Similarity, the mean alignment score is also increasing. That means that protein pairs with high Jaccard Similarities are more likely to have similar amino acid sequences, structures and interaction interfaces. Therefore, the first potential reason for high Jaccard Similarity is high interface similarity, and a protein with complementary interface to them becomes their common neighbor. For example, if a protein C interacts with A which has high Jaccard Similarity with B, then C may also interact with B because the interface complementarity between C and A leads to the possibility that the interfaces of C and B are also complementary.

#### 2.2.2. Gene Duplication

Another reason for choosing Jaccard Similarity is from gene duplication (Zhang, 2003; Dehal and Boore, 2005). In the process of evolution, genes may produce new products: new proteins, which may retain many of the properties of the original ones and consequently preserve many interacting partners.

We find several proteins that are recorded as the products of gene duplication events from (PhylomeDB), and then generate several organisms' PPI networks containing them from (STRING). We delete links with confidence <0.7 in the PPI networks to ensure the reliability. The Jaccard Similarities and alignment scores of these protein pairs are shown in Table 2. We can see that protein pairs from gene duplication events have high Jaccard Similarities and alignment scores. Because the products of gene duplication have similar amino acid sequence, which leads to the similarity of their structures and interaction interfaces. As a result, they share a large proportion of interaction neighbors in PPI network, i.e., they have high Jaccard Similarities. Therefore, the second potential reason for a high Jaccard Similarity protein pair is that they are products of gene duplication, and a protein that interact with one of them is likely to interact with the other one. For example, if a protein C interacts with A which is a product of gene duplication to B, then C may interact with B too.

**Table 2:** Comparison of alignment scores of protein pairs produced by gene duplication events and Jaccard Similarities of them.

**PPI networks**	**Protein A**	**Protein B**	**Jaccard similarity**	**Alignment score**
*Arabidopsis thaliana*	PIN7	PIN3	0.7913834	0.84202454
*Caenorhabditis elegans*	abu-7	abu-6	0.70384985	0.874429224
*Escherichia coli* K12 MG1655	rhsA	rhsB	1	0.896167247
	rhsD	rhsA	0.8021216	0.711538462
	rhsD	rhsB	0.7920696	0.746896552
*Mus musculus*	Camk2a	Camk2g	0.8977933	0.766917293
	Camk2g	Camk2d	0.91231686	0.833955224
	Camk2a	Camk2d	0.9082842	0.829457364
	Camk2b	Camk2g	0.9142654	0.827648115
	Camk2a	Camk2b	0.9103021	0.765567766
	Camk2b	Camk2d	0.9255441	0.834545455
*Homo sapiens*	RAB5B	RAB5C	0.9483445	0.748
	RAB5A	RAB5C	0.78506744	0.764940239
*Saccharomyces cerevisiae*	TDH2	TDH1	0.87267643	0.885542169
	TDH3	TDH2	0.8513397	0.963855422
	TDH3	TDH1	0.81701237	0.88253012

*PPI networks are from (STRING), and the evidence of gene duplication events are from (PhylomeDB)*.

To sum up, proteins with complementary interfaces interact with each other, and proteins with similar interfaces share interacting partners; the similarity of gene duplication products leads to sharing interacting neighbors. And Jaccard Similarity can reflect the interface similarity between protein pairs as well as the similarity between gene duplication products. Based on that, we propose a basic assumption: the more similar proteins are, the more likely they are to share more interacting partners, rather than interacting with each other. This is the basis for the index *Sim* we will propose in section 2.4. In the following subsections, in order to further verify the rationality of using Jaccard Similarity for similarity measure, we will compare the performances of our index *Sim* with indices using other similarity measures on two types of random networks and real PPI networks.

### 2.3. Two Types of Random Network Models

From the two aspects of interface complementarity and gene duplication, we simulate the linkage mechanism of PPI networks and generate two types of random network models.

#### 2.3.1. Random Network Based on Complementarity of Interfaces

Based on the hypothesis that interacting proteins have complementary interfaces and proteins with similar interfaces may share more interaction partners, we propose the first random network model. We assume that there are *d* types of interface pairs in a PPI network. Each node of a PPI network is represented by a *d*-dimension vector *X*.

(6) $$\begin{array}{l} X=\left(x_{1},x_{2},\ldots ,x_{d}\right)\\ \text{Where }x_{i}=\{\begin{array}{ll} 0, & \text{ if X have no interface }i^{+}\text{and }i^{-},\\ 1, & \text{ if X have interface }i^{+},\\ 2, & \text{ if X have interface }i^{-},\\ 3, & \text{if X have both interface }i^{+}\text{ and }i^{-}. \end{array}i=1,2,\ldots ,d. \end{array}$$

**Figure 5:**
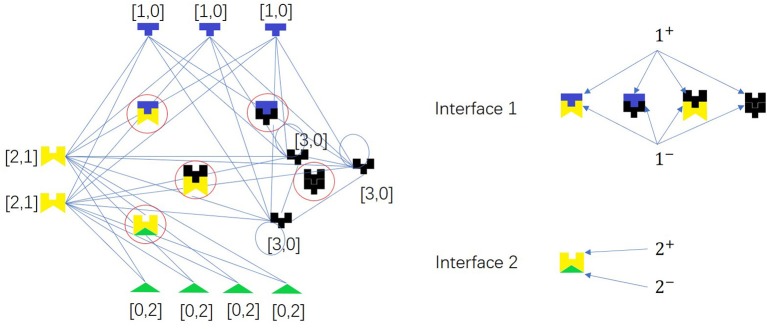
A network model for PPI network based on complementarity of interfaces. The colored pieces are proteins, and the ones in the circles are protein complexes. Node pair with high Jaccard Similarity (especially for same-color nodes) may have similar interaction interfaces. There are two types of interface pairs, i.e., *d* = 2.

Two proteins can interact with each other if and only if they have complementary interfaces *i*^+^ and *i*^−^ of one pair *i*, respectively, i.e., there is a link between *X* = (*x*_1_, *x*_2_, …, *x*_*d*_) and *Y* = (*y*_1_, *y*_2_, …, *y*_*d*_) if and only if ∃*i* ∈ {1, 2, …, *d*}, s.t. (*x*_*i*_, *y*_*i*_) ∈ {(1, 2), (2, 1), (1, 3), (3, 1), (2, 3), (3, 2), (3, 3)}. There is an example as shown in Figure 5.

For each node *X*, we set *x*_*i*_ = 0, 1, 2, 3 with probabilities *p*_1_, *p*_2_, *p*_3_ and *p*_4_, where *i* = 1, 2, …, *d*. According to different values of *p*_1_, *p*_2_, *p*_3_, *p*_4_ and the number of nodes *n*, we generate six random networks named test_graph1, test_graph2, …, and test_graph6.

#### 2.3.2. Random Network Based on Gene Duplication

**Figure 6:**
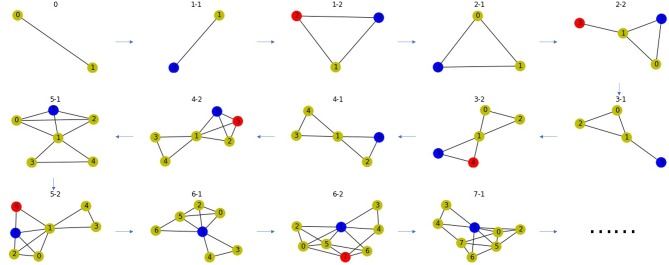
A network model for PPI network based on gene duplication. Subfigure 0 is the original network, the blue node in each subfigure is the node chosen for splitting, the red node is the new node after splitting. The blue and red node have high Jaccard Similarity in the PPI network.

Based on the principle of gene duplication, we propose the second random network model. We randomly select a node *u* from a PPI network, split it into two nodes *u* and *v*, and make *v* replicate the same links as *u* with probability *p*. Repeat this process many times to obtain the final network. This process may end up with a disconnected network, so we take the largest connected component to ensure the connectivity of the network. Network-based approaches can only be used on connected networks. If the network is disconnected, links can be predicted on each connected component, respectively. In order to explain the linking mechanism of this random network, it is only necessary to take any one of the connected components, and it is more reasonable to select the largest connected component, because the minimum one may be trivial, i.e., it only has one node or one link. There is an example as shown in Figure 6.

We generate six random networks named Eva1, Eva2, …, and Eva6 with different values of parameter *p* and *n*. In the next section, We will propose a link prediction index using Jaccard Similarity, and compare our index with the indices using other similarity measures through experiments on these two random network models.

### 2.4. Link Prediction

For other complex networks, similarity measure is usually used directly for link prediction (Katz, 1953; Newman, 2001; Barabâsi et al., 2002; Adamic and Adar, 2003; Lü et al., 2009; Zhou et al., 2009; Wang et al., 2015). After the analysis in the previous subsections, we use Jaccard Similarity (Jaccard, 1912) to measure the interface similarity and paralogy, not the linkage likelihood. In other words, two proteins with high similarity do not necessarily interact with each other, i.e., they do not have to be linked in the PPI network. Therefore, we do not predict links between high Jaccard Similarity node pairs since similar interfaces do not lead to their interactions but complementary interfaces.

Recall that in sections 2.2.1 and 2.2.2, we show that Jaccard Similarity can reflect the interface and evolutionary similarity of protein pairs. In other words, we assume that the potential reason of high Jaccard Similarity between two nodes in a PPI network is that their interfaces are similar or they are the products of gene duplication events. Therefore, the two proteins with high Jaccard Similarity do not need to interact with each other, but share a large proportion of interacting partners, i.e., their proportion of common neighbors in all their neighbors. Therefore, high Jaccard Similarity does not necessarily increase the likelihood of their interaction, but rather the likelihood that non-common partners will become common partners. For example, if the link between Gria1 and Camk2d is missed in Figure 3, we can predict it according to that Gria1 may be a common neighbor of Camk2d and Camk2g since they are of high Jaccard Similarity. In other words, if node *i*'s neighbors and *j* are very similar (high Jaccard Similarity), then *i* may becomes a common neighbor of them, i.e., we predict that there is a link between *i* and *j*. Based on that, we propose a link prediction index named *Sim*.

(7) $$\begin{array}{l} Sim_{ij}=\underset{v\in \Gamma \left(j\right)}{\sum }AJ_{vi}+\underset{u\in \Gamma \left(i\right)}{\sum }AJ_{uj} \end{array}$$

Where *A* is the adjacency matrix and *J*_*uj*_ is the Jaccard Similarity between *u* and *j* which is defined in Equation (5).

*Sim* index can also be expressed in a matrix form:

(8) $$\begin{array}{l} Sim=AJ+JA \end{array}$$

where *A* is the adjacency matrix and *J* is the similarity matrix (i.e., *J*_*u,v*_ is the Jaccard Similarity between *u* and *v*).

**Figure 7:**
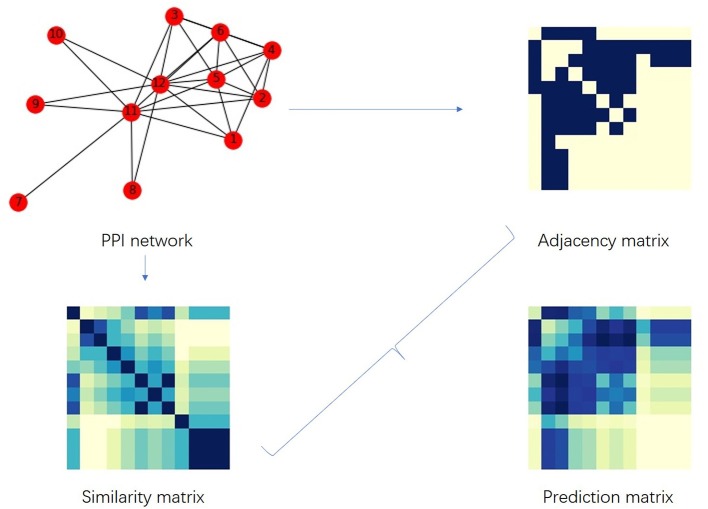
The flow chart of the link prediction algorithm *Sim*. The darker the color of the small squares in the prediction matrix, the more likely the value is 1.

Figure 7 shows the flow of our proposed algorithm. PPI network *G* is the input, we first obtain the adjacency matrix *A* of *G*, then calculate the similarity matrix *J*, and then bring them into Equation (8) to calculate the link score matrix (prediction matrix: *P*) as the output. After ranking *P*_*ij*_ from large to small, we can set a threshold or the number of predictions (i.e., *k*) to distinguish linkage from non-linkage predictions.

*LO* is defined as *AS*, unlike it, *Sim* index is defined as *AJ*+*JA*. *LO* only considers the contribution of the similarity between *i*'s neighbors and *j*. There is no problem for directed networks. But for undirected networks, such as PPI networks, there will be a contradiction that *LO*_*ij*_ ≠ *LO*_*ji*_. We take this into account when designing *Sim* index. Because our target networks PPI networks are undirected networks, we define *Sim* = *AS*+*SA*. *Sim* can be regarded as a special case of *LO* for undirected networks when *S* = *J*. The similarity matrix *S* in *LO* is obtain by solving an optimization problem (Equation 2), but the optimum may not guarantee the best performance of link prediction. We also consider the cases of *J* = *CN, PA* and *RA* and get several indices: *SimCN, SimPA*, and *SimRA*.

**Figure 8:**
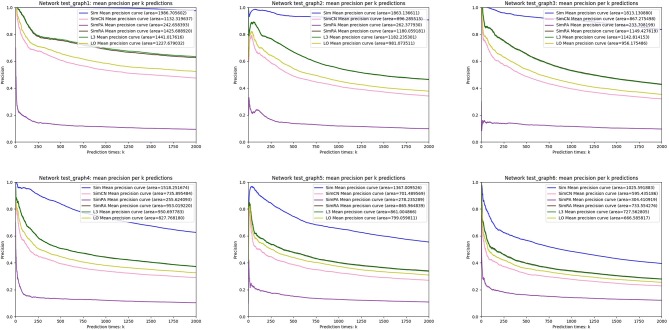
Precision curves on random networks based on complementarity of interfaces.

**Figure 9:**
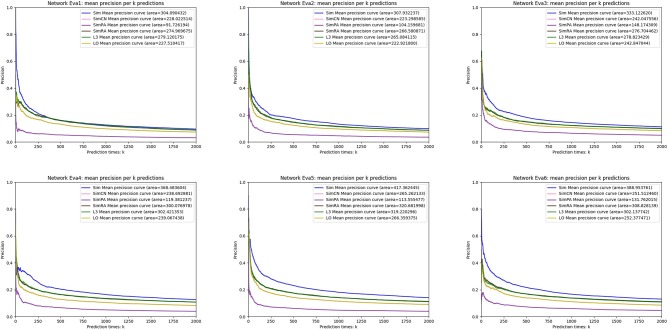
Precision curves on random networks based on gene duplication.

We simulated *Sim, SimCN, SimPA, SimRA*, *L*3, and *LO* on the two types of random network models we mentioned in the previous subsection, and compared their performances as shown in Figures 8, 9. We use Precision curve to evaluate the indices. Ten-fold cross validation is executed to avoid over fitting.

From the simulation results, we can see that our index *Sim* not only outperforms indices using other similarities (*SimCN, SimPA*, and *SimRA*), but also outperforms baselines (*L*3 and *LO*) on the two types of random network models. Can the excellent performance of *Sim* in random network models be reproduced in real networks? In the next section we will discuss in detail through experiments on real PPI networks.

## 3. Results and Discussion

### 3.1. Data

In order to verify the performance of our method on real PPI networks, we select PPI networks of different organisms from several independent data sets: (HINT), (BIOGRID), (STRING), (PrePPI), and (Pajek). Some PPI networks are weighted graphs. The weights of the links represent the confidence. We normalize the weights and delete the links with weights <0.7 to avoid false positives. For comparison, we also consider some non-PPI networks, including social networks, transportation networks from (KONECT) and (Pajek), random networks: Erdős-Rényi graphs (Erdős and Rényi, 1960) and Watts Strogatz small-world graphs (Watts and Strogatz, 1998). The name of the network “erdos_renyi_n500_p04” represents the value of the parameter: *n, p* = 500, 0.04; “watts_strogatz_n500_k20_p10” represents *n, k, p* = 500, 20, 0.10. The sources of these networks and their statistical characteristics are shown in Table 3. Abbreviations in Table 3: clus., Asso., Av-de., Hete., and Dens. are average clustering coefficient, assortativity coefficient, average degree, degree heterogeneity, and link density, respectively.

**Table 3:** Statistical characteristics and sources of PPI networks.

**Type**	**Network name**	**#Nodes**	**#Edges**	**Clus**.	**Asso**.	**Av-de**.	**Hete**.	**Dens**.	**#Rings**
PPI	*A. thaliana* (HINT)	5,646	23,410	0.06	−0.207	8.293	9.467	0.001	595
	*Arabidopsis* (STRING)	447	3,675	0.366	0.22	16.443	1.988	0.037	0
	*B. subtilis* (HINT)	625	1,152	0.084	0.132	3.686	3.868	0.006	378
	BIOGRID-PF (BIOGRID)	1,227	2,508	0.014	−0.011	4.088	2.751	0.003	63
	BIOGRID-RN (BIOGRID)	4,185	6,666	0.098	−0.266	3.186	37.598	0.001	57
	*C. elegans* (HINT)	4,809	12,234	0.038	−0.095	5.088	5.861	0.001	327
	*E. coli* (STRING)	450	7,743	0.245	0.091	34.413	1.403	0.076	0
	*D. melanogaster* (HINT)	8,293	30,182	0.016	−0.057	7.279	3.521	0.001	393
	*E. coli* (HINT)	2,176	3,655	0.052	0.01	3.359	2.497	0.002	1270
	hi-ii-14 (Kovács et al., 2019)	4,298	13,868	0.052	−0.208	6.453	7.102	0.002	518
	hi-iii (Kovács et al., 2019)	5,604	23,322	0.068	−0.186	8.323	7.86	0.001	322
	hi-tested (Kovács et al., 2019)	3,727	9,433	0.025	−0.216	5.062	5.737	0.001	445
	Human (STRING)	436	4,024	0.342	0.027	18.459	2.301	0.042	0
	Marina (STRING)	450	8,925	0.272	0.208	39.667	1.354	0.088	0
	Mouse (STRING)	444	4,802	0.38	0.02	21.631	2.378	0.049	0
	Oryza (STRING)	440	6,899	0.347	0.089	31.359	1.999	0.071	0
	PrePPI-human2011 (PrePPI)	7,863	23,779	0.073	−0.162	6.048	9.728	0.001	621
	*S. cerevisiae* (HINT)	5,315	23,203	0.102	−0.131	8.731	3.964	0.002	1,138
	*S. pombe* (HINT)	1,488	2,583	0.045	−0.137	3.472	5.265	0.002	407
	Yeast (STRING)	427	4,570	0.26	0.129	21.405	1.673	0.05	0
	Yeasts (Pajek)	2,361	7,182	0.13	−0.085	6.084	2.763	0.003	536
Other	Bible (KONECT)	1,773	9,131	0.721	−0.049	10.3	4.011	0.006	0
	Chicago (KONECT)	1,467	1,298	0	−0.505	1.77	3.059	0.001	0
	erdos_renyi_n500_p04	500	4,910	0.038	−0.024	19.64	1.048	0.039	0
	erdos_renyi_n500_p06	500	7,513	0.06	−0.009	30.052	1.032	0.06	0
	erdos_renyi_n500_p08	500	9,993	0.08	0.014	39.972	1.025	0.08	0
	erdos_renyi_n500_p10	500	12,488	0.101	−0.003	49.952	1.018	0.1	0
	Euroroad (KONECT)	1,174	1,417	0.017	0.127	2.414	1.242	0.002	0
	Infectious (KONECT)	410	2,765	0.456	0.226	13.488	1.388	0.033	0
	Netscience (Pajek)	1,461	2,742	0.694	0.462	3.754	1.849	0.003	0
	watts_strogatz_n500_k20_p10	500	5,000	0.526	−0.006	20	1.005	0.04	0
	watts_strogatz_n500_k40_p10	500	10,000	0.545	−0.01	40	1.002	0.08	0
	watts_strogatz_n500_k60_p10	500	15,000	0.549	0.011	60	1.002	0.12	0
	watts_strogatz_n500_k80_p10	500	20,000	0.561	0.007	80	1.001	0.16	0

### 3.2. Comparison With Other Network-Based Methods

**Figure 10:**
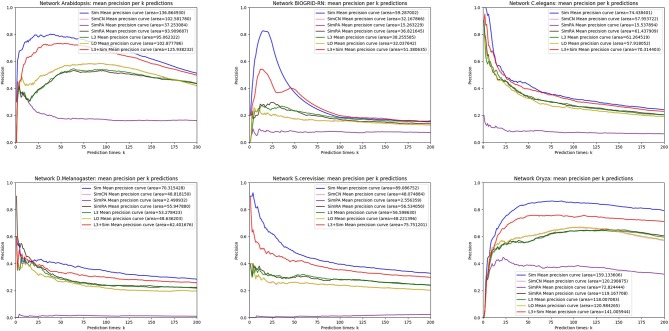
Precision curves for six PPI networks with *Sim* outperforming the other indices.

**Figure 11:**
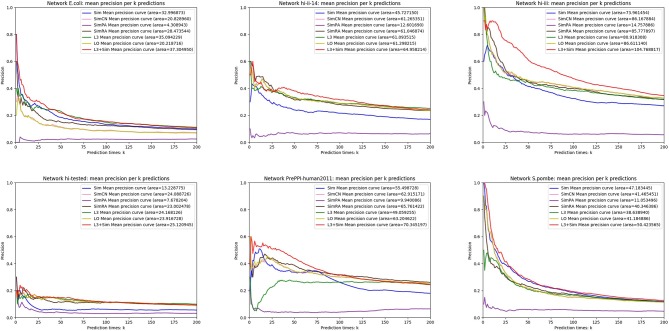
Precision curves for six PPI networks with *L*3 + *Sim* outperforming the other indices.

**Figure 12:**
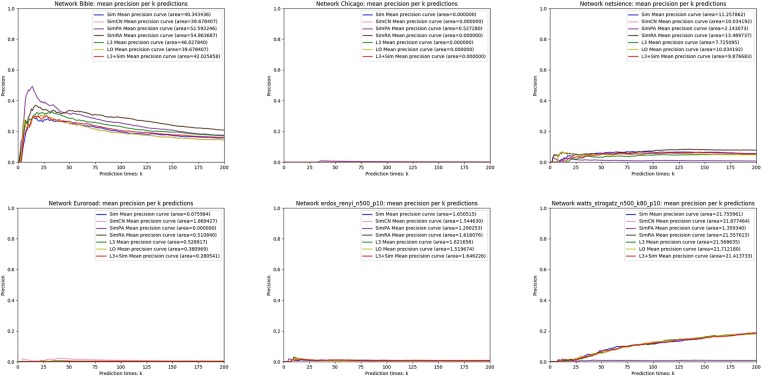
Precision curves for six non-PPI networks with *Sim* and *L*3 + *Sim* not outperforming the other indices.

In this section, we use seven link prediction indices: *L*3, *LO, Sim, SimCN, SimPA, SimRA*, and *L*3 + *Sim* to predict links of PPI networks in Table 3. *L*3 + *Sim* is the integration of *L*3 and *Sim*. The precision curves are shown in Figures 10–12. Each precision curve is the average of 10 precision curves from 10-fold cross validations. Abscissa is the number of predictions (i.e., *k*), ordinate is the precision of top-*k* predictions (i.e., true positive rate), “area” is the area under the precision curve. The larger the area, the better the performance of the method within *k* predictions. We take the maximum value of *k* as 200, because a large value of *k* has no practical significance. For example, for a PPI network with 1,000 nodes and 10,000 links, if 500 links are missing, then we need to predict 500 real links among all the possible 490,500 node pairs, which is a very difficult task. The precision of random prediction is ~0.001. Obviously, we will not make 490,500 positive predictions. Very large *k* will lead to very low precision, and there is no guidance for biological experiments due to the high cost.

**Figure 13:**
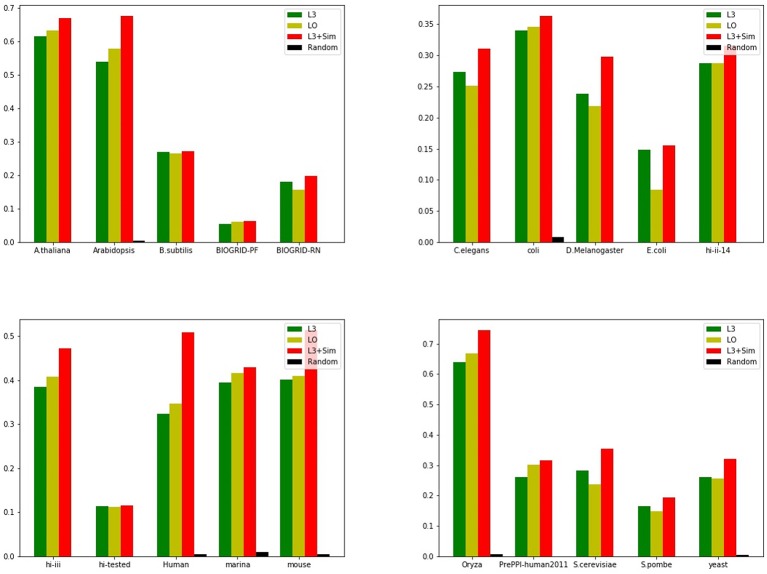
Precision of Top 100 predictions of *L*3 + *Sim* for all PPI networks in Table 3 compared with baselines. In terms of precision, the integration index *L*3 + *Sim* is not only many times higher than the random method, but also surpasses the baselines.

We can see that, for PPI networks in Figure 10, *Sim* outperforms all indices. But for PPI networks in Figure 11, *Sim* does not outperform *L*3 or *LO*. However, the integration of *Sim* and *L*3 can outperform both *L*3 and *LO* for almost all PPI networks. As can be seen from Figure 12, *Sim* and *L*3 + *Sim* do not outperform baselines for non-PPI networks. Especially, we notice that almost all indices fail for networks “Chicago” and “Euroroad.” The precision is almost equal to or even lower than guess. This means that none of these indices reflect the self-organization mechanism of these two non-PPI networks. By sharp contrast, as can be seen from Figure 13, our index *L*3 + *Sim* is not only much more accurate than guess, but also outperforms *L*3 and *LO* for almost all PPI networks. To sum up, we come to two conclusions:

1. *L*3 + *Sim* improves the link prediction performance of *L*3 for PPI networks but not for non-PPI networks.
2. The precision of *L*3 + *Sim* is tens of times to thousands of times higher than guess for PPI networks but not for non-PPI networks.

## 4. Conclusion

In this paper, we propose a network-based link prediction method *Sim* for PPI networks. This index is designed from two perspectives: the complementarity of protein interaction interfaces and gene duplication. We propose two types of random network models to simulate these two linkage generation mechanisms of PPI networks. We explain the reasons of using of Jaccard Similarity in *Sim* by sequence alignment, and they are confirmed by experiments on two types of random networks.

In order to improve the robustness of prediction, we proposed the integration of *L*3 and *Sim*: *L*3 + *Sim*. Experiments on independent data sets show that *Sim* outperforms other indices for several of these PPI networks. However, the integration method *L*3 + *Sim* is always superior to the baselines: *L*3 and *LO*. For the precision of top 100 predictions, *L*3 + *Sim* is 15–20% higher than *L*3 and *LO* on average. We only use the information of PPI network itself to propose a method that can pick out protein pairs which are more likely to interact with each other form a huge number of candidates, and provide them for high-throughput experiments. Like other network-based methods, the disadvantage of our method is that we can not predict the link between two nodes in different connected components. In the future research, we can integrate the information of nodes to network-based methods to make up for the shortage.

## Data Availability Statement

The data and source code of Sim is freely available from: https://github.com/wingroy001/L3Sim.

## Author Contributions

YC and WW: conceptualization. YC: data curation, formal analysis, investigation, resources, software, visualization, and writing—original draft. XG and YC: funding acquisition, writing—review, and editing. YC and JF: methodology. YC, WW, and XG: project administration. WW and XG: supervision. YC and JL: validation.

### Conflict of Interest

The authors declare that the research was conducted in the absence of any commercial or financial relationships that could be construed as a potential conflict of interest.

## References

[B1] Adamic L. A. Adar E. (2003). Friends and neighbors on the web. Soc. Netw. 25, 211–230. 10.1016/S0378-8733(03)00009-1

[B2] AlQuraishi M. (2019). Alphafold at casp13. Bioinformatics 35, 4862–4865. 10.1093/bioinformatics/btz422 31116374 PMC6907002

[B3] An J.-Y. Zhang L. Zhou Y. Zhao Y.-J. Wang D.-F. (2017). Computational methods using weighed-extreme learning machine to predict protein self-interactions with protein evolutionary information. J. Cheminform. 9:47. 10.1186/s13321-017-0233-z 29086182 PMC5561767

[B4] Backstrom L. Leskovec J. (2011). Supervised random walks: predicting and recommending links in social networks, in Proceedings of the Fourth ACM International Conference on Web Search and Data Mining (Hong Kong: ACM), 635–644. 10.1145/1935826.1935914

[B5] Barabâsi A.-L. Jeong H. Néda Z. Ravasz E. Schubert A. Vicsek T. (2002). Evolution of the social network of scientific collaborations. Phys. A Stat. Mech. Appl. 311, 590–614. 10.1016/S0378-4371(02)00736-7

[B6] Cannistraci C. V. Alanis-Lobato G. Ravasi T. (2013). From link-prediction in brain connectomes and protein interactomes to the local-community-paradigm in complex networks. Sci. Rep. 3:1613. 10.1038/srep01613 23563395 PMC3619147

[B7] Chen F. Mackey A. J. Stoeckert C. J. Jr. Roos D. S. (2006). Orthomcl-db: querying a comprehensive multi-species collection of ortholog groups. Nucleic Acids Res. 34, D363–D368. 10.1093/nar/gkj123 16381887 PMC1347485

[B8] Clauset A. Moore C. Newman M. E. (2008). Hierarchical structure and the prediction of missing links in networks. Nature 453:98. 10.1038/nature06830 18451861

[B9] Cuatrecasas P. (1970). Protein purification by affinity chromatography derivatizations of agarose and polyacrylamide beads. J. Biol. Chem. 245, 3059–3065. 5432796

[B10] Dehal P. Boore J. L. (2005). Two rounds of whole genome duplication in the ancestral vertebrate. PLoS Biol. 3:e314. 10.1371/journal.pbio.0030314 16128622 PMC1197285

[B11] Dick K. Green J. R. (2018). Reciprocal perspective for improved protein-protein interaction prediction. Sci. Rep. 8:11694. 10.1038/s41598-018-30044-1 30076341 PMC6076239

[B12] Erdős P. Rényi A. (1960). On the evolution of random graphs. Publ. Math. Inst. Hung. Acad. Sci. 5, 17–60.

[B13] Fields S. Song O.-K. (1989). A novel genetic system to detect protein-protein interactions. Nature 340:245. 10.1038/340245a0 2547163

[B14] Huang L. Liao L. Wu C. H. (2016). Inference of protein-protein interaction networks from multiple heterogeneous data. EURASIP J. Bioinform. Syst. Biol. 2016:8. 10.1186/s13637-016-0040-2 26941784 PMC4761017

[B15] Huang L. Liao L. Wu C. H. (2017). Evolutionary analysis and interaction prediction for protein-protein interaction network in geometric space. PLoS ONE 12:e0183495. 10.1371/journal.pone.0183495 28886027 PMC5590856

[B16] Jaccard P. (1912). The distribution of the flora in the alpine zone. New Phytol. 11, 37–50. 10.1111/j.1469-8137.1912.tb05611.x

[B17] Katz L. (1953). A new status index derived from sociometric analysis. Psychometrika 18, 39–43. 10.1007/BF02289026

[B18] Kovács I. A. Luck K. Spirohn K. Wang Y. Pollis C. Schlabach S.. (2019). Network-based prediction of protein interactions. Nat. Commun. 10:1240. 10.1038/s41467-019-09177-y 30886144 PMC6423278

[B19] Lichtenwalter R. N. Lussier J. T. Chawla N. V. (2010). New perspectives and methods in link prediction, in Proceedings of the 16th ACM SIGKDD International Conference on Knowledge Discovery and Data Mining (ACM), 243–252. 10.1145/1835804.1835837

[B20] Lin T.-W. Wu J.-W. Chang D. T.-H. (2013). Combining phylogenetic profiling-based and machine learning-based techniques to predict functional related proteins. PLoS ONE 8:e75940. 10.1371/journal.pone.0075940 24069454 PMC3777923

[B21] Lü L. Jin C.-H. Zhou T. (2009). Similarity index based on local paths for link prediction of complex networks. Phys. Rev. E 80:046122. 10.1103/PhysRevE.80.046122 19905405

[B22] Lü L. Zhou T. (2011). Link prediction in complex networks: a survey. Phys. A Stat. Mech. Appl. 390, 1150–1170. 10.1016/j.physa.2010.11.027

[B23] MacBeath G. Schreiber S. L. (2000). Printing proteins as microarrays for high-throughput function determination. Science 289, 1760–1763. 10.1126/science.289.5485.1760 10976071

[B24] Moresco J. J. Carvalho P. C. Yates I. I. I. J. R. (2010). Identifying components of protein complexes in *C. elegans* using co-immunoprecipitation and mass spectrometry. J. Proteomics 73, 2198–2204. 10.1016/j.jprot.2010.05.008 20546956 PMC3279190

[B25] Muscoloni A. Abdelhamid I. Cannistraci C. V. (2018). Local-community network automata modelling based on length-three-paths for prediction of complex network structures in protein interactomes, food webs and more. bioRxiv 346916 10.1101/346916

[B26] Needleman S. B. Wunsch C. D. (1970). A general method applicable to the search for similarities in the amino acid sequence of two proteins. J. Mol. Biol. 48, 443–453. 10.1016/0022-2836(70)90057-4 5420325

[B27] Newman M. E. (2001). Clustering and preferential attachment in growing networks. Phys. Rev. E 64:025102. 10.1103/PhysRevE.64.025102 11497639

[B28] Norel R. Lin S. L. Wolfson H. J. Nussinov R. (1994). Shape complementarity at protein-protein interfaces. Biopolymers 34, 933–940. 10.1002/bip.360340711 8054472

[B29] Pech R. Dong H. Li L. Y. Ye Y. Tao Z. (2019). Link prediction via linear optimization. Phys. A 528:121319 10.1016/j.physa.2019.121319

[B30] Peng J. Xu J. (2011). Raptorx: exploiting structure information for protein alignment by statistical inference. Proteins Struct. Funct. Bioinform. 79, 161–171. 10.1002/prot.23175 21987485 PMC3226909

[B31] Planas-Iglesias J. Bonet J. García-García J. Marín-López M. A. Feliu E. Oliva B. (2013). Understanding protein-protein interactions using local structural features. J. Mol. Biol. 425, 1210–1224. 10.1016/j.jmb.2013.01.014 23353828

[B32] Symeonidis P. Iakovidou N. Mantas N. Manolopoulos Y. (2013). From biological to social networks: link prediction based on multi-way spectral clustering. Data Knowl. Eng. 87, 226–242. 10.1016/j.datak.2013.05.008

[B33] Tsoka S. Ouzounis C. A. (2000). Prediction of protein interactions: metabolic enzymes are frequently involved in gene fusion. Nat. Genet. 26:141. 10.1038/79847 11017064

[B34] Wang P. Xu B. Wu Y. Zhou X. (2015). Link prediction in social networks: the state-of-the-art. Sci. China Inform. Sci. 58, 1–38. 10.1007/s11432-015-5403-x

[B35] Wang Y. You Z. Li X. Chen X. Jiang T. Zhang J. (2017a). Pcvmzm: using the probabilistic classification vector machines model combined with a zernike moments descriptor to predict protein-protein interactions from protein sequences. Int. J. Mol. Sci. 18:1029. 10.3390/ijms18051029 28492483 PMC5454941

[B36] Wang Y.-B. You Z.-H. Li X. Jiang T.-H. Chen X. Zhou X.. (2017b). Predicting protein-protein interactions from protein sequences by a stacked sparse autoencoder deep neural network. Mol. Biosyst. 13, 1336–1344. 10.1039/C7MB00188F 28604872

[B37] Watts D. J. Strogatz S. H. (1998). Collective dynamics of ‘small-world' networks. Nature 393:440. 10.1038/30918 9623998

[B38] You Z.-H. Li X. Chan K. C. (2017). An improved sequence-based prediction protocol for protein-protein interactions using amino acids substitution matrix and rotation forest ensemble classifiers. Neurocomputing 228, 277–282. 10.1016/j.neucom.2016.10.042

[B39] Zhang J. (2003). Evolution by gene duplication: an update. Trends Ecol. Evol. 18, 292–298. 10.1016/S0169-5347(03)00033-8

[B40] Zhao C. Zang Y. Quan W. Hu X. Sacan A. (2017). Hiv1-human protein-protein interaction prediction based on interface architecture similarity, in 2017 IEEE International Conference on Bioinformatics and Biomedicine (BIBM) (Hong Kong: IEEE), 97–100. 10.1109/BIBM.2017.8217632

[B41] Zhou T. Lü L. Zhang Y.-C. (2009). Predicting missing links via local information. Eur. Phys. J. B 71, 623–630. 10.1140/epjb/e2009-00335-8

